# Supplementary material for: Unraveling the transcriptomic effects of leucine supplementation on muscle growth and performance in basketball athletes
Source: PLoS One. 2025 Jan 24;20(1):e0316603. doi: 10.1371/journal.pone.0316603 (PMC11761164; doi:10.1371/journal.pone.0316603)
Supplement: Attachment — Submitted filename: Response to reviewers.docx [file pone.0316603.s002.docx]

Journal Requirements:

When submitting your revision, we need you to address these additional requirements.

1. Please ensure that your manuscript meets PLOS ONE's style requirements, including those for file naming. The PLOS ONE style templates can be found at https://journals.plos.org/plosone/s/file?id=wjVg/PLOSOne_formatting_sample_main_body.pdf and https://journals.plos.org/plosone/s/file?id=ba62/PLOSOne_formatting_sample_title_authors_affiliations.pdf

R: Thanks, we have revised this.

2. We suggest you thoroughly copyedit your manuscript for language usage, spelling, and grammar. If you do not know anyone who can help you do this, you may wish to consider employing a professional scientific editing service. The American Journal Experts (AJE) (https://www.aje.com/) is one such service that has extensive experience helping authors meet PLOS guidelines and can provide language editing, translation, manuscript formatting, and figure formatting to ensure your manuscript meets our submission guidelines. Please note that having the manuscript copyedited by AJE or any other editing services does not guarantee selection for peer review or acceptance for publication. Upon resubmission, please provide the following: ● The name of the colleague or the details of the professional service that edited your manuscript ● A copy of your manuscript showing your changes by either highlighting them or using track changes (uploaded as a *supporting information* file) ● A clean copy of the edited manuscript (uploaded as the new *manuscript* file)

R: This article has been edited by a professional native speaker

3. In the online submission form, you indicated that “The data underlying the results presented in the study are available from corresponding author upon request.”.

All PLOS journals now require all data underlying the findings described in their manuscript to be freely available to other researchers, either 1. In a public repository, 2. Within the manuscript itself, or 3. Uploaded as supplementary information. This policy applies to all data except where public deposition would breach compliance with the protocol approved by your research ethics board. If your data cannot be made publicly available for ethical or legal reasons (e.g., public availability would compromise patient privacy), please explain your reasons on resubmission and your exemption request will be escalated for approval.

R: The raw data of sequencing have been attached as supplementary material within the manuscript.

4. PLOS requires an ORCID iD for the corresponding author in Editorial Manager on papers submitted after December 6th, 2016. Please ensure that you have an ORCID iD and that it is validated in Editorial Manager. To do this, go to ‘Update my Information’ (in the upper left-hand corner of the main menu), and click on the Fetch/Validate link next to the ORCID field. This will take you to the ORCID site and allow you to create a new iD or authenticate a pre-existing iD in Editorial Manager.

R: Thanks, I have provided the ORCID ID.

5. Please include your full ethics statement in the ‘Methods’ section of your manuscript file. In your statement, please include the full name of the IRB or ethics committee who approved or waived your study, as well as whether or not you obtained informed written or verbal consent. If consent was waived for your study, please include this information in your statement as well.

R: Thanks, we have revised these.

6. Please ensure that you refer to Figure 6 in your text as, if accepted, production will need this reference to link the reader to the figure.

R: Thank you! We have referenced Figure 6 in the text.

Review Comments to the Author

Reviewer #1: **Linguistically, be sure to have a native English person review the article.

R: Thanks, we have polished the language of this article by a professional English native speaker.

**In any part of the article where we use abbreviations, we must first bring the full vocabulary and then use the abbreviations.

R: Thanks, we have revised these, we have provided the full vocabulary of the abbreviations when they firstly occurred.

**In the text of the article, you mentioned GAP, but no explanation was given.

R: it denotes that while leucine's general benefits for athletic performance are well-documented, there is insufficient or inconclusive data on its specific impact or application to basketball players. Therefore, our research seeks to fill this gap by exploring how leucine supplementation could influence performance metrics, recovery, and muscle function specifically in the context of basketball. We have added the explanation of this in the main text.
**Determine the reruns of the feeding protocol and what the rest of the subjects' diet was like.

R: For the feeding protocol, During the exercise test, all the participants followed a controlled diet consisting of prepackaged meals and snacks that provided 1.5 times their resting energy expenditure (REE). The diet included 1.2 grams of protein per kilogram of body weight per day (g/kg/d), 4 g/kg/d of carbohydrates, and the remainder of calories from fat, ensuring a balanced intake tailored to their energy needs. Participants were instructed to drink only water in addition to the provided meals and snacks, standardizing hydration and nutrient intake throughout the testing period to control for variables that could influence exercise performance.

For the drinking, participants were given a 0.25 g·kg-1 carbohydrate solution mixed with 16 ounces of water (total 0.50 g·kg-1) 30 minutes before and immediately after exercise. The independent variable was the addition of 25 mg·kg-1 leucine powder to the test drinks before and after exercise (total 50 mg·kg-1) for the experimental group.

**In the discussion, you mentioned that studies, but you mentioned one study.

R: Thanks, we have revised this. ‘Studies have highlighted the pivotal role of leucine in stimulating muscle protein synthesis via the mTOR signaling pathway. Crozier et al. (2005) demonstrated that leucine activates mTOR, promoting downstream effectors like S6K1 and 4E-BP1, which enhance translation initiation, crucial for muscle growth and repair. Norton et al. (2009) showed that leucine supplementation after resistance exercise increases phosphorylation of mTOR and its downstream targets, resulting in enhanced protein synthesis, supporting its role in exercise-induced hypertrophy. Additionally, Anthony et al. (2000) found that leucine acts as a nutrient signal to the mTOR pathway, even in the absence of other amino acids, promoting muscle protein synthesis independently of other dietary factors. “

**Mention the strengths and weaknesses of the work.

R: I have added the discussion of the strengths and weaknesses of the work in the discussion part. “However, the study has some limitations, such as the lack of statistically significant improvements in other performance metrics and short duration, which may have restricted its ability to capture the full benefits of leucine supplementation. The focus on short-term effects and the absence of data on leucine’s interactions with other nutrients also limit the broader applicability of the findings.”

Reviewer #2: Thank you for providing me the opportunity of manuscript entitle: “Unraveling the Transcriptomic Effects of Leucine Supplementation on Muscle Growth and Performance in Basketball Athletes”. The title is interesting , however some concerns are raised or following points are suggested:

Major grammatical revisions are required in all parts of the manuscript.
1- Abstract
The following phrase or sentences need rewording:
- ” we measure their exercise performance like 282 feet sprint”
- subject self-rating at practices as baseline
- Subsequently, the same exercise performances were recorded as above
- but there still have promoted tendency
-doses of leucine need to be included.
Conclusion is not based on the results.
The tense of most sentences should become past

R: Thanks for your suggestions, we have revised our abstract according to your advice.
2- introduction
- The physiological rationale selecting variables of sport performance according to physiological base is not clear and must be mentioned.

R: “The selected variables—282 feet sprint, free throws, and three-point field goals—reflect key physiological and skill demands in basketball. The sprint tests anaerobic capacity and fast-twitch muscle performance crucial for quick, high-intensity movements, while free throws and three-point shots measure precision, muscle coordination, and endurance needed for accurate shooting. Together, these variables capture both the physical and technical aspects of basketball performance.” We have included these in the introduction.
- The rationale for selecting every gene must be mentioned.

R: Yes, the rational of selecting genes have been included in the introduction.
- What is the necessity of this research while the effects of leucine on hypertrophy as well as performance have been investigated in several previous studies.

R: Although the general benefits of leucine for athletic performance are well-established, there is a lack of research specifically examining its effects in basketball, particularly in terms of how leucine supplementation may impact performance metrics, recovery, and muscle function within this sport.
3- Method
- The method of recruiting participants is not clear.

R: thanks, we have revised this. "In total, 20 men's basketball players (aged 18–23 years) were recruited from a university basketball team for this study. All participants held a Level 1 basketball athlete qualification. Volunteers were recruited through advertisements. This study received approval from the university ethics committee."
- The method of sample size calculation is not clear.

R: Thanks, we have revised this, “In this study, data were processed using GraphPad Prism 8.0.1 software. A two-way ANOVA was employed to analyze the data, allowing for the examination of the effects of two independent variables and their interaction on the dependent variable. Following the ANOVA, Tukey’s multiple comparison test was applied to identify specific group differences. A p-value of less than 0.05 was considered statistically significant.”
- This sentence is not proper for method” The body weight, height and Body mass index (BMI), which accounts for both height and weight, was not significantly different between the 2 groups. “

R: We have rewritten this sentence. “The body weight and height of participants were measured to ensure comparability between the two groups. No significant differences were found in these parameters between the groups.”
- Ethics approval is necessary for this manuscript while it was mentioned an N/A.

R: we have received the ethics approval from university ethics committee, the statement has been updated in the main text.

- The phrase “The measures measured included timed sprints and shooting accuracy,
“ needs rewording

R: We have revised this. “The assessments included timed sprints and shooting accuracy.”
- Tissue sampling method is not available.

R: we have revised this. “In this study, muscle samples from the biceps were collected from each group after a 28-day experimental period for RNA sequencing (RNA-seq) analysis. Six replicate muscle samples were obtained from each group. The biceps samples were carefully excised under sterile conditions and immediately flash-frozen in liquid nitrogen to preserve RNA integrity. They were then stored at -80°C until RNA extraction. This process ensured high-quality samples for subsequent RNA-seq analysis.”
- Physical performance tests must be explained in details.

R: Thanks, we have added more details about this in methods section.
4- Discussion
- All measured variables findings must be disussed individually.

R: Thanks, we have revised this, we have discussed all the measure variables findings in the discussion. “ Notably, a significant improvement in the 282-foot sprint performance following leucine supplementation, with sprint times decreasing from 17.4 ± 0.9 to 16.2 ± 0.9 seconds, compared to a minimal change in the control group (P = 0.034). This suggests that leucine may enhance speed and agility. In contrast, while there was a trend towards improvement in three-point shooting accuracy (from 10.2 ± 1.1 to 11.1 ± 1.4) and free throw performance (from 8.0 ± 0.8 to 8.4 ± 0.8) in the leucine group, these changes were not statistically significant compared to the control group. This indicates that while leucine might have a positive effect on shooting performance, the results were not conclusive. Additionally, subjective ratings of mental and physical well-being showed a slight improvement in the leucine group (from 7.3 ± 0.9 to 7.9 ± 0.7), whereas the control group remained relatively stable (from 7.1 ± 0.9 to 7.3 ± 0.9). Although this improvement was not statistically significant (P > 0.05), it also suggests a potential benefit of leucine on overall well-being.”
- Limitations must be included.

R: I have added the limitation of the work in the discussion part. “However, the study has some limitations, such as the lack of statistically significant improvements in other performance metrics and short duration, which may have restricted its ability to capture the full benefits of leucine supplementation. The focus on short-term effects and the absence of data on leucine’s interactions with other nutrients also limit the broader applicability of the findings.”
