# Supplementary material for: Unraveling the transcriptomic effects of leucine supplementation on muscle growth and performance in basketball athletes
Source: PLoS One. 2025 Jan 24;20(1):e0316603. doi: 10.1371/journal.pone.0316603 (PMC11761164; doi:10.1371/journal.pone.0316603)
Supplement: Attachment — Submitted filename: Response to Reviewer.docx [file pone.0316603.s003.docx]

Reviewer #2: Thank you for providing me the opportunity of manuscript entitle: “Unraveling the Transcriptomic Effects of Leucine Supplementation on Muscle Growth and Performance in Basketball Athletes”. The title is interesting , however some concerns are raised or following points are suggested:

Still there are Major grammatical problems, and most of required revisions have not been considered.

are required in all parts of the manuscript.

1-Abstract

The following phrase or sentences need rewording:

“ we total recruit 20 basketball player and randomly divided into two groups”

R： we have revised this sentence. “ In this study, a total of 20 basketball players were recruited and randomly divided into two groups.”

-subject self-rating at practices as baseline

-Subsequently, the same exercise performances were recorded as above

R: This sentence has been revised. “Prior to leucine supplementation, baseline exercise performance, including a 282-foot sprint, free throws, three-point field goals, and self-rated practice assessments, was evaluated. Participants were then provided with a functional drink, either with or without leucine supplementation (50 mg/kg body weight), for 28 days. Subsequently, the same exercise performance metrics were reassessed.”

-but there still have promoted tendency

R: we have revised this. “For other exercise performance metrics, no significant differences were observed (P > 0.05); however, a trend toward improvement was noted.”

Conclusion is not based on the results.

R: We have revised the summary part in abstract. “In summary, Leucine supplementation improved exercise performance, significantly reducing sprint times and showing trends of improvement in three-point field goals, free throws, and self-rated well-being. Transcriptome analysis identified 3,658 differentially expressed genes enriched in pathways related to immune response, cytokine production, and cell adhesion. Weighted Gene Co-expression Network Analysis (WGCNA) highlighted a key module strongly associated with muscle growth traits, with functional enrichment in cytoskeletal pathways. qPCR validation confirmed upregulation of cytoskeleton-related genes, supporting transcriptomic findings. These results suggest that leucine enhances muscle adaptation by regulating cytoskeletal dynamics, offering molecular insights into its role in improving athletic performance.”

The tense of most sentences should change past

R: the tense of the sentences have been checked.

2-introduction

again following questions have not been answered.

- The physiological rationale selecting variables of sport performance according to physiological base is not clear and must be mentioned. Several sentences have been included without any reference.

R: Thanks, The following sentences have declared the physiological rational of selecting sport performance. “ The selected performance variables—282-foot sprint, free throws, and three-point field goals—were chosen based on their relevance to the physiological and skill demands of basketball. The sprint test was designed to assess anaerobic capacity, which is critical for high-intensity, short-duration efforts, as well as the activation and performance of fast-twitch muscle fibers required for rapid and explosive movements during gameplay. Free throws and three-point shots were included to evaluate precision, muscle coordination, and endurance, all of which are necessary for maintaining shooting accuracy under physical and mental fatigue.”

We have included reference in the miantext as well, thanks for suggestions.

-The rationale for selecting every gene must be mentioned.

R: The following sentences have declare the rationale of selected genes. And we have added the references. “The genes of interest were selected based on their critical roles in maintaining cytoskeletal integrity, regulating muscle contraction, and promoting tissue repair, all of which are essential for muscle adaptation to physical stress and high-intensity activity. Specifically, α-tubulin and β-tubulin are key components of microtubules, which provide structural support and facilitate intracellular transport. CK18 and CK8, as intermediate filaments, contribute to cellular integrity and resilience under mechanical stress. Vimentin is involved in maintaining cell shape and stability during dynamic cellular processes. Actin-binding proteins, such as cofilin, gelsolin, and profilin, regulate actin filament assembly and disassembly, crucial for muscle contraction and cytoskeletal remodeling. MAP1, MAP2, and MAP4 are microtubule-associated proteins that modulate microtubule stability and dynamics, supporting cellular structure during muscle activity. E-cadherin and N-cadherin are adhesion proteins that play pivotal roles in cell-cell adhesion, necessary for tissue integrity and coordinated muscle function. These genes collectively represent fundamental processes such as actin and microtubule dynamics, cell adhesion, and cytoskeletal stability, which leucine supplementation may influence to enhance muscle strength, function, and recovery.”

3-Method

-The method of sample size calculation is not clear.

R: Thanks, we have declared this. “ The sample size for this study was determined using a power analysis to ensure sufficient statistical power for detecting changes in performance metrics (e.g., sprint times, free-throw accuracy) between the experimental and control groups. A two-tailed analysis with a power of 80% (β = 0.2) and a significance level of 0.05 (α = 0.05) was conducted.”

-Ethic committee number is not available.

R: This experiment has been approved by the Ethics Committee of South China Normal University (SCNU-SPT-2023-031).

-This sentence is not proper properly stated” The body weight, height and Body mass index (BMI), which accounts for both height and weight, was not significantly different between the 2 groups. “

R: We have revised this. “ Participants' body weight and height were measured to ensure comparability between the two groups, and no significant differences were detected.”

-Ethics approval number is necessary for this manuscript .

R: This experiment has been approved by the Ethics Committee of South China Normal University (SCNU-SPT-2023-031).

-Regarding diet, when did they eat food before testing?

R: “Participants consumed their last prepackaged meal or snack approximately 2-3 hours before each exercise test to ensure adequate digestion and energy availability while minimizing the risk of gastrointestinal discomfort during performance assessments. This timing was standardized across all participants to maintain consistency.” We have included this in the Methods.

-Did you consider muscle hypertrophy (size as an important mediatory factor for performance)?

R: Muscle hypertrophy was not directly measured in this study; however, its potential role as a mediatory factor for basketball performance was considered. Instead of focusing on muscle size, we emphasized functional outcomes, such as sprint performance, shooting accuracy, and subjective well-being, as these are more directly linked to basketball performance. Additionally, the transcriptomic analysis highlighted cytoskeletal and contractile pathways, which are integral to muscle adaptation and function, suggesting an indirect impact on hypertrophy. Future studies could incorporate direct measures of muscle size, such as imaging or biopsy, to better understand its mediatory role in basketball performance.

4-Discussion

-All measured variables findings must be discussed individually.

We appreciate the reviewer’s valuable feedback and agree that discussing each measured variable individually will enhance the clarity and depth of our findings. Below, we provide a detailed discussion of all measured variables, incorporating both performance and molecular insights:
