# Supplementary material for: Unraveling the transcriptomic effects of leucine supplementation on muscle growth and performance in basketball athletes
Source: PLoS One. 2025 Jan 24;20(1):e0316603. doi: 10.1371/journal.pone.0316603 (PMC11761164; doi:10.1371/journal.pone.0316603)
Supplement: Attachment — Submitted filename: Response to Reviewer.docx [file pone.0316603.s004.docx]

Journal Requirements:

Please review your reference list to ensure that it is complete and correct. If you have cited papers that have been retracted, please include the rationale for doing so in the manuscript text, or remove these references and replace them with relevant current references. Any changes to the reference list should be mentioned in the rebuttal letter that accompanies your revised manuscript. If you need to cite a retracted article, indicate the article’s retracted status in the References list and also include a citation and full reference for the retraction notice.

R:Dear editor, we have double-checked the references and make a revision.

Reviewer #2: please consider minor grammatical revisions.

Note: please look at the conclusion, is it a conclusion of your manuscript?

4.4 Conclusion

We have now thoroughly discussed each measured variable and its

implications for athletic performance and molecular adaptations. By

addressing these findings individually, we aim to provide a clearer

understanding of leucine’s potential benefits and limitations within the context

of basketball performance. We hope this revision meets the reviewer’s

expectations and clarifies our study’s contributions.

R: Sorry, its a mistake. We have revised the conclusion as below. “4.4 Conclusion

These results supported the hypothesis that leucine supplementation could optimize muscle function by enhancing cytoskeletal dynamics, thereby improving athletic performance, particularly in sports that require high-intensity, anaerobic efforts. Given its effects on muscle adaptation and recovery, leucine could also be explored as a supplement to reduce fatigue and accelerate recovery in athletes, promoting sustained performance in both training and competition in the future.
